# Patient characteristics, symptoms, and urodynamic parameters associated with detrusor contraction duration in women

**DOI:** 10.3389/fruro.2023.1173506

**Published:** 2023-05-25

**Authors:** Connie N. Wang, Albert Ha, Doreen E. Chung

**Affiliations:** Department of Urology, Columbia University Irving Medical Center, New York, NY, United States

**Keywords:** detrusor, voiding dysfunction, lower urinary tract obstruction, bladder, detrusor contractility, detrusor contraction duration

## Abstract

**Background:**

There is a lack of understanding of the clinical significance of detrusor contraction duration (DCD) measured on urodynamic studies (UDS). We aimed to identify patient characteristics, presenting symptoms and urodynamic parameters associated with DCD in women.

**Methods:**

Using a single-institution database of UDS (2015-2019), 405 female patients with measurable detrusor contractions were identified. Baseline characteristics, presenting symptoms and UDS parameters were analyzed. Bladder outlet obstruction (BOO) was characterized using the Blaivas-Groutz nomogram. Wilcox Rank Sum Tests were used for descriptive statistics, and a univariable generalized linear model conforming to a gamma distribution was used.

**Results:**

Median age was 65 years (IQR 52-75), BMI was 27.5 kg/m2 (IQR 23.9-31.1) and DCD was 90 seconds (IQR 57-124). On univariable analysis, degenerative disc disease (β = -17.9, p = 0.02), pelvic radiation (β = -31.91, p = 0.04), and stress incontinence (β = -14.11, p = 0.03) were associated with reduced DCD. Black race was associated with longer DCD (β = 22.92, p = 0.01). Analysis of UDS parameters revealed a significant increase in DCD per unit increase of bladder capacity (β = 0.08, p<0.001), detrusor pressure (Pdet) at maximum flow (Qmax) (β = 0.96, p<0.001), and maximum Pdet (β = 1.2, p<0.001). In contrast, a significant decrease in DCD was noted per unit increase in Qmax (β = -1.43, p<0.001). Finally, mild (β = 34.4, p<0.001), moderate (β = 72.52, p<0.001), and severe (β = 64.6, p<0.001) BOO were all associated with increased DCD.

**Conclusions:**

Median DCD in women is 90 seconds. Longer DCD is associated with greater degree of BOO, higher maximum Pdet, Pdet at Qmax, and bladder capacity. Disc disease, irradiation and stress incontinence are associated with reduced DCD. Further studies are needed to evaluate the predictive value of DCD in women.

## Introduction

Detrusor underactivity (DU) is a common syndrome of lower urinary tract dysfunction that remains poorly understood, difficult to quantify, and challenging to treat in clinical practice (1). The International Continence Society (ICS) has defined DU as “a contraction of reduced strength and/or duration, resulting in prolonged bladder emptying and/or failure to achieve complete bladder emptying within a normal time span’’ (2). Nevertheless, a variety of terminologies and conflicting definitions remain in the literature to describe this syndrome of voiding dysfunction that may include features of sensation of incomplete bladder emptying, retention and/or elevated post void residuals, and reduced urinary flow rate. Diagnosis of DU is further complicated by the overlap of these signs and symptoms in the presence of bladder outlet obstruction (BOO), and determination of true etiology of symptoms requires interpretation of urodynamic studies (UDS)

Although UDS parameters that describe detrusor contraction strength, such as maximum detrusor pressure, maximum flow rate, and detrusor pressure at maximum flow, have been well characterized, detrusor contraction duration (DCD) is rarely reported on in the voiding dysfunction literature or in clinical practice. DCD is defined as time elapsed from beginning of increased detrusor pressure during volitional voiding to time of decrease of detrusor pressure to pre-micturition pressure. Previous studies have suggested that persistence of detrusor contraction, as measured by DCD on UDS, may represent another means of assessing voiding function, as a correlate of symptom severity as well as a potentially useful predictor of response to therapies for lower urinary tract symptoms (LUTS) (3, 4).However, to date, scant data regarding DCD have been reported in the literature and is limited to small series of mostly male patients (3–6).There is no current standardization of “normal” DCD ranges, which prevents uniformity in UDS interpretation and voiding dysfunction diagnosis

In this study, we sought to characterize the spectrum of DCD in a large cohort of female patients undergoing UDS evaluation for LUTS, and to elucidate associations between DCD, patient characteristics, presenting symptoms, and other UDS parameters.

## Materials and methods

### Data source

In this study, we utilized data from a prospectively maintained single-institution UDS database (2015-2019) of patients who presented with LUTS. Clinical parameters within the database included patient demographics, presenting voiding symptoms, and UDS parameters, extracted from electronic medical records. Male patients and female patients with involuntary detrusor contractions were excluded from analysis to minimize confounding. Duplicate UDS studies on a single patient were excluded.

All patients underwent multichannel urodynamic evaluation using Laborie (Toronto, Ontario, Canada) equipment. All UDS were evaluated by a single fellowship-trained urologist specializing in voiding dysfunction following the recommendations of the International Continence Society (ICS) Good Urodynamics Practice standards. Bladder pressure was monitored using a dual lumen 7 French urethral catheter inserted into the bladder. Abdominal pressure was recorded using a standard rectal balloon catheter. Slow-fill cystometry was performed at 15-30 ml/min. All contractions, whether voluntary or involuntary, were noted along with all filling and voiding UDS parameters. Definitions were consistent with those by the ICS (2). Parameters analyzed included bladder capacity, bladder compliance, maximum flow (Qmax), maximum detrusor pressure (Pdet), detrusor pressure at maximum flow (Pdet at Qmax), voided volume, and DCD. DCD was measured in seconds by hand from original UDS tracings, beginning at initial increase in Pdet and ending at return to baseline Pdet, without a change in abdominal pressure (PAbd). Detrusor contractions were considered voluntary if they occurred following permission to void. Involuntary contractions were defined as a nonvolitional increase in Pdet during filling with or without sense of urgency, and without an associated increase in PAbd.

### Study covariates

Baseline patient characteristics such as age, body mass index (BMI), race, presenting symptoms, and additional medical history such as smoking history and the presence of diabetes were reported. Presenting symptoms included frequency, urgency, stress urinary incontinence (SUI), urge incontinence, decreased stream, incomplete emptying and retention, straining, and nocturia. UDS parameters, as described above, included cystometric bladder capacity, bladder compliance, Qmax, Pdet at Qmax, voided volume, maximum Pdet, and DCD. Bladder outlet obstruction (BOO) was defined by criteria set out by Blaivas and Groutz, defined as Q_max_ <12 mL/s with P_det_ >20 cmH_2_O (7).

### Statistical analysis

Patient characteristics were compared using descriptive statistics with Wilcox Rank Sum Tests. A univariable generalized linear model conforming to a gamma distribution was used to analyze the relationship between patient characteristics, presenting symptoms, and UDS parameters with DCD. All tests were two-sided with a statistical significance of p ≤ 0.05. All analysis was performed using R version 4.00 (R Foundation for Statistical Computing, Vienna, Austria). This retrospective review was approved by the Columbia University Institutional Review Board.

## Results

We identified 405 female patients with measurable, voluntary detrusor contractions. Patient demographics can be found in **Table 1**. Median age was 65 years (IQR 52-75) and median BMI was 27.5 kg/m (2) (IQR 23.9-31.1). Patients in this cohort self-reported their race as 73.1% White, 21.5% Black, 3.2% Asian/Pacific Islander, and 2.2% Other. The most prevalent presenting symptoms included urgency incontinence (74.6%), urgency (73.3%), nocturia (68.4%), and frequency (63.5%). Median DCD was 90 seconds (IQR 57-124) (**Table 1**).

**Table 1:** Patient Demographics, Presenting Symptoms, and Urodynamic Parameters.

Patient Characteristics	Median (IQR)
Age (years)	65 (23)
BMI (kg/m2)	27.5 (7.19)
Race	N (%)
White	296 (73.1)
Black	87 (21.5)
Asian/Pacific Islander	13 (3.2)
Other	9 (2.2)
Smoking	75 (18.5)
Diabetes Mellitus	83 (20.5)
Spinal Cord Injury	4 (1)
Stroke	13 (3.2)
Degenerative Disk Disease	68 (16.8)
Coronary Artery Disease	32 (7.9)
Neurogenic Bladder	43 (10.6)
Post-radiation	8 (2)
Presenting Symptoms	N (%)
Frequency	257 (63.5)
Urgency	297 (73.3)
Urge Incontinence	302 (74.6)
Stress Incontinence	226 (55.8)
Incomplete Emptying/Retention	204 (50.4)
Straining	76 (18.8)
Decreased Stream	77 (19)
Nocturia	277 (68.4)
UDS Parameters	Median (IQR)
Capacity	404 (221.75)
Compliance	100 (150.3)
Qmax	12 (14)
PdetQmax	25 (20)
Detrusor Contraction Duration	90 (67)
Maximum Pdet	31 (22)
VV	286.5 (294.25)
BOO	N (%)
None	115 (28.4)
Mild	249 (62.4)
Moderate	19 (4.8)
Severe	22 (5.5)

On univariable analysis, degenerative disc disease (β = -17.9, p = 0.02), pelvic radiation (β **=** -31.91, p = 0.04), and stress incontinence (β = -14.11, p = 0.03) were associated with reduced DCD (**Table 2**). In contrast, black race was associated with longer DCD (β = 22.92, p = 0.01). When analyzing UDS parameters, a significant increase in DCD was noted per unit increase of bladder capacity (β **=** 0.08, p <0.001), Pdet at Qmax (β = 0.96, p <0.001), and maximum Pdet (β = 1.2, p <0.001). In contrast, a significant decrease in DCD was noted per unit increase in Qmax (β = -1.43, p <0.001). Although no association was noted in compliance, a trend of increased DCD was noted per unit increase of voided volume (β = 0.03, p = 0.08).

**Table 2:** Univariable Regression Analysis of Female Patient Characteristics, Symptoms, and Urodynamic Parameters with DCD.

Univariable		
Covariates	Beta [95% CI]	P-value
Patient Characteristics		
Age	-0.21 [-0.63, 0.2]	0.29
BMI	-0.69 [-1.6, 0.36]	0.15
Race		
White	–	–
Black	22.92 [6.89, 41.26]	0.01
Asian/Pacific Islander	37.14 [-0.16, 96.19]	0.11
Other	-1.32 [-31.86, 51.34]	0.95
Smoking	-1.33 [-16.37, 15.92]	0.87
Diabetes Mellitus	-2.91 [-17.32, 13.38]	0.71
Spinal Cord Injury	61.2 [-8.99, 228.74]	0.24
Stroke	-19.97 [-43.41, 16.48]	0.17
Degenerative Disk Disease	-17.9 [-31.67, -2.23]	0.02
CAD	-12.42 [-30.81, 11.34]	0.24
Neurogenic Bladder	4.49 [-14.54, 28.27]	0.68
Post-radiation	-31.91 [-55.97, 10.82]	0.04
Presenting Symptoms		
Frequency	0.66 [-12.72, 13.44]	0.92
Urgency	-4.94 [-20.26, 8.99]	0.51
Urge Incontinence	-5.61 [-21.33, 8.56]	0.46
Stress Incontinence	-14.11 [-27.33, -1.37]	0.03
Incomplete Emptying or Retention	1.64 [-10.96, 14.24]	0.80
Straining	13.1 [-3.12, 31.93]	0.14
Decreased Stream	-3.25 [-17.97, 13.56]	0.68
Nocturia	5.46 [-8.26, 18.37]	0.42
UDS Parameters		
Capacity	0.08 [0.04, 0.12]	<0.001
Compliance	0.04 [-0.03, 0.12]	0.30
Qmax	-1.43 [-1.88, -0.93]	<0.001
Pdet at Qmax	0.96 [0.54, 1.41]	<0.001
Maximum Pdet	1.2 [0.79, 1.63]	<0.001
Voided Volume	0.03 [-0.001, 0.06]	0.08
BOO Index for Females		
None		
Mild	34.4 [22.84, 45.72]	<0.001
Moderate	72.52 [38.11, 121.36]	<0.001
Severe	64.6 [33.78, 106.98]	<0.001

Finally, mild (β = 34.4, p <0.001), moderate (β = 72.52, p <0.001), and severe (β = 64.6, p <0.001) BOO were all significantly associated with increased DCD (**Table 2**).

## Discussion

Although prevalence of DU in clinical series has been reported to be between 12-45% of older women undergoing urodynamic evaluation for non-neurogenic LUTS, it remains challenging to recognize and treat (1). Reduced DCD during volitional voiding contributes to the clinical signs and symptoms of DU voiding dysfunction, but this UDS parameter has not been well studied. Few papers have examined this parameter in men and even fewer in women (**Table 3**).

**Table 3:** Reported DCD Values in Existing Literature.

Study	Study Cohort	Presenting symptoms	Reported DCD Data
Kaplan et al. (1996) (3)	120 patients (63 men, 57 women)	Moderate or severe AUA-SS*	Mean DCD in men with moderate AUA-SS: 109.3 ± 17.0 sec., severe AUA-SS: 149.9 ± 21.4 sec. Mean DCD in women with moderate AUA-SS: 82.5 ± 14.7 sec., severe AUA-SS: 69.4 ± 12.0 sec.
Turner et al. (1998) (8)	91 men	Unspecified, untreated LUTS	Data not available
Ameda et al. (1998) (5)	58 men	Unspecified LUTS	Mean DCD in patients with mild AUA-SS: 116.7 ± 34.0 sec., moderate AUA-SS: 102.7 ± 61.9 sec., severe AUA-SS: 89.2 ± 44.4 sec. Mean DCD in obstructed patients: 111.6 ± 53.7 sec., non-obstructed patients: 91.5 ± 41.5 sec. Mean DCD in patients with good contractility: 94.3 ± 49.2 sec., poor contractility: 115.5 ± 43.3 sec.
Kaplan et al. (2000) (4)	85 men	Unspecified, untreated LUTS	Mean DCD: 107.6 ± 28.6 sec.
Aganovic et al. (2004) (6)	102 men	Proven benign prostatic enlargement	Mean DCD in non-obstructed patients with low grade of detrusor contraction: 95.9 ± 26.5 sec. vs. in obstructed patients with low grade of detrusor contraction: 104.5 ± 21.1 sec., (p>0.05) Mean DCD in non-obstructed patients with high grade of detrusor contraction: 85.3 ± 25.2 sec. vs. in obstructed patients with high grade of detrusor contraction: 108.4 ± 32.8 sec., (p<0.005)
Present study	408 women	LUTS including frequency, urgency, stress urinary incontinence, urge urinary incontinence, decreased stream, incomplete emptying, straining, and nocturia	Median DCD: 90 sec. (IQR 57-124)

*AUA-SS, American Urological Association symptom scores; sec, seconds; LUTS, lower urinary tract symptoms.

Kaplan et al. was the first group to study DCD in 1996 by reviewing UDS records of 120 consecutive patients (63 men and 57 women) who had moderate or severe American Urological Association symptom scores. They found that while there was no correlation between UDS parameters and symptoms in women, in men, increasing DCD was correlated with both overall symptom severity and increasing obstructive symptoms. This study also found a correlation between increasing DCD and urodynamic evidence of BOO in men (3).

Subsequently, Turner et al. evaluated the voiding symptoms and UDS of 91 men with untreated LUTS before and after initiation of medical therapy for BOO and found that DCD was the only UDS parameter that correlated significantly with severity of voiding symptoms as assessed by the International Prostate Symptom Score (8). Kaplan et al.’s group performed a similar analysis of UDS of 85 men with LUTS before and after medical therapy for BOO and found that a high baseline DCD (greater than or equal to 90 seconds) was correlated with therapeutic response to medical therapy (4). Taken together, these studies’ findings suggest that DCD may represent a useful clinical UDS parameter to predict the efficacy of therapy of BOO.

However, other data is conflicting. Ameda et al. studied UDS of 58 consecutive men with LUTS to determine if DCD was a useful parameter for characterizing BOO (5). In contrast to Kaplan et al.’s findings, this group did not observe a difference in DCD between patients with mild, moderate or severe AUA symptom scores. Furthermore, there was no difference in DCD in patients who were obstructed compared to those who were not obstructed. Meanwhile, Aganovic et al. assessed urodynamic measurements of 102 men with benign prostatic enlargement and found that DCD was longer in patients with higher levels of obstruction, as defined according to the Schafer nomogram. However, in their cohort, DCD was not associated with symptom severity (as defined by the International Prostate Symptom Score).

Recent reviews of the existing literature on DU reveal that while this is a common phenomenon in both men and women undergoing UDS evaluation for non-neurogenic LUTS, this syndrome of voiding dysfunction remains poorly studied and managed in clinical practice, given lack of standardized definitions and multiple possible etiologies with varying symptom presentations (1, 9). While there has been increased interest and research in this topic in recent years, existing diagnostic criteria of DU remain poorly validated and only address detrusor contraction strength, while neglecting speed and persistence of bladder contractions (9). DCD represents a simple, reproducible UDS parameter that merits further investigation for inclusion into future formulas to diagnosis DU and/or predict response to therapies for DU and BOO.

Our study is the first to study DCD in a very large cohort of women and the first to focus specifically on DCD and its relationship to other UDS parameters. It is also the first study to examine DCD from a large number of UDS records from a prospectively maintained database. The median DCD in this population, 90 seconds, was similar to the mean DCD observed in female patients in Kaplan et al.’s study (69 - 83 seconds). Additionally, as other authors have previously described, we also found that patients with BOO of any degree had significantly increased DCD compared to patients without BOO.

Our findings that history of degenerative disc disease and pelvic radiation were associated with reduced DCD merits further analysis with these patient subgroups. However, the clear correlation of presenting symptom of stress incontinence with decreased DCD suggests that decreased outlet resistance may contribute to shorter DCD. Conversely, parameters associated with increased outlet resistance, such as increased bladder capacity, Pdet at Qmax, and maximum Pdet, were found to be correlated with increase in DCD, further supporting a correlation between longer DCD and BOO.

Limitations of this study include single-institution source of data, lack of age stratification, lack of data on patients’ medication usage for LUTS, and risk of confounding due to the overlap in certain UDS findings and symptoms associated with DU and BOO. Furthermore, use of the Blaivas-Groutz criteria to define BOO in women may have overestimated obstruction compared to other urodynamic definitions of BOO (10). Nevertheless, this represents by far the largest cohort of patients and UDS records studied to date to evaluate the relationship between DCD and patient demographics, presenting symptoms and other UDS parameters. These results add to our understanding of the spectrum of DCD in women presenting for LUTS and can guide future evaluations of DU diagnosis and clinical management. The predictive relationship between DCD, Pdet, BOO, and bladder emptying symptoms requires further analysis. Without standardization of the terminology, measurements, and diagnostic criteria of DU, further research into the etiologies of this syndrome and investigations of various treatment efficacies will not be possible.

## Conclusion

This is one of the first studies to examine the clinical significance of the UDS parameter of DCD. Median DCD in our cohort of female patients was approximately 1.5 minutes. Longer DCD is associated with greater degree of BOO, higher maximum Pdet, Pdet at Qmax, and bladder capacity. History of disc disease, radiation and presenting symptom of stress incontinence are associated with reduced DCD. These findings suggest that stronger detrusor contractility and greater outlet resistance may contribute to longer DCD. Further studies are needed to establish causality and the overall contribution of DCD to bladder emptying.

## Data availability statement

The original contributions presented in the study are included in the article/supplementary materials. Further inquiries can be directed to the corresponding author.

## Ethics statement

The studies involving human participants were reviewed and approved by Columbia University Institutional Review Board. Written informed consent for participation was not required for this study in accordance with the national legislation and the institutional requirements.

## Author contributions

CW, AH, and DC contributed equally to the design, analysis and interpretation of the study. CW contributed to drafting of the work and AH and DC contributed to revising the final manuscript. All author provide approval for publication of the content and agree to be accountable for all aspects of the work in ensuring that questions related to the accuracy or integrity of any part of the work are appropriately investigated and resolved. All authors contributed to the article and approved the submitted version.
